# Effects of sample preservation methods and duration of storage on the performance of mid-infrared spectroscopy for predicting the age of malaria vectors

**DOI:** 10.1186/s13071-022-05396-3

**Published:** 2022-08-06

**Authors:** Jacqueline N. Mgaya, Doreen J. Siria, Faraja E. Makala, Joseph P. Mgando, John-Mary Vianney, Emmanuel P. Mwanga, Fredros O. Okumu

**Affiliations:** 1 Environmental Health and Ecological Science Department, Ifakara Health Institute, P.O. Box 53, Ifakara, Tanzania; 2 School of Life Science and Bioengineering, The Nelson Mandela African Institute of Science and Technology, P.O. Box 447, Arusha, Tanzania; 3 School of Public Health, Faculty of Health Sciences, University of the Witwatersrand, Johannesburg, South Africa; 4 Institute of Biodiversity, Animal Health, and Comparative Medicine, University of Glasgow, Glasgow, G12 8QQ UK

**Keywords:** Malaria, Vector control, Sample handling, *An.arabiensis*, Age-grading, Machine learning and infrared spectroscopy

## Abstract

**Background:**

Monitoring the biological attributes of mosquitoes is critical for understanding pathogen transmission and estimating the impacts of vector control interventions on the survival of vector species. Infrared spectroscopy and machine learning techniques are increasingly being tested for this purpose and have been proven to accurately predict the age, species, blood-meal sources, and pathogen infections in *Anopheles* and *Aedes* mosquitoes. However, as these techniques are still in early-stage implementation, there are no standardized procedures for handling samples prior to the infrared scanning. This study investigated the effects of different preservation methods and storage duration on the performance of mid-infrared spectroscopy for age-grading females of the malaria vector, *Anopheles arabiensis.*

**Methods:**

Laboratory-reared *An. arabiensis* (*N* = 3681) were collected at 5 and 17 days post-emergence, killed with ethanol, and then preserved using silica desiccant at 5 °C, freezing at − 20 °C, or absolute ethanol at room temperature. For each preservation method, the mosquitoes were divided into three groups, stored for 1, 4, or 8 weeks, and then scanned using a mid-infrared spectrometer. Supervised machine learning classifiers were trained with the infrared spectra, and the support vector machine (SVM) emerged as the best model for predicting the mosquito ages.

**Results:**

The model trained using silica-preserved mosquitoes achieved 95% accuracy when predicting the ages of other silica-preserved mosquitoes, but declined to 72% and 66% when age-classifying mosquitoes preserved using ethanol and freezing, respectively. Prediction accuracies of models trained on samples preserved in ethanol and freezing also reduced when these models were applied to samples preserved by other methods. Similarly, models trained on 1-week stored samples had declining accuracies of 97%, 83%, and 72% when predicting the ages of mosquitoes stored for 1, 4, or 8 weeks respectively.

**Conclusions:**

When using mid-infrared spectroscopy and supervised machine learning to age-grade mosquitoes, the highest accuracies are achieved when the training and test samples are preserved in the same way and stored for similar durations. However, when the test and training samples were handled differently, the classification accuracies declined significantly. Protocols for infrared-based entomological studies should therefore emphasize standardized sample-handling procedures and possibly additional statistical procedures such as transfer learning for greater accuracy.

**Graphical Abstract:**

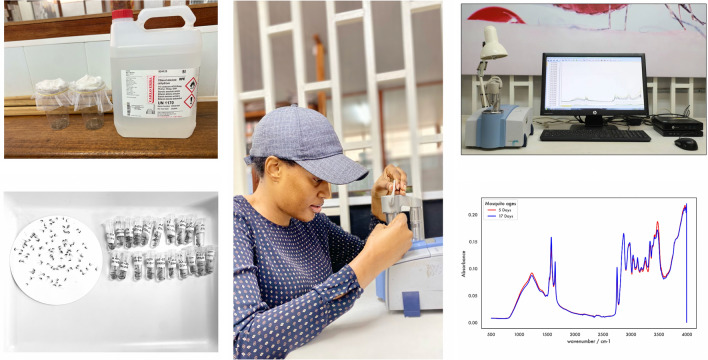

## Background

In 2020, malaria deaths increased by 12% as compared to 2019, reaching a global total of ~ 627,000, nearly all of which occurred in sub-Saharan Africa [1]. The main methods for controlling the disease currently include improved case management with artemisinin-based combination therapies (ACTs) and vector control with either insecticide-treated mosquito nets (ITNs) or indoor residual spraying (IRS). For over 2 decades, these tools have been the mainstay of malaria control, contributing significantly to the reductions in cases and deaths [2].

In addition to case management and vector control, the World Health Organization (WHO), in their 2016–2030 Global Technical Strategy, recommended that improved surveillance should also be included as a core component of malaria control [3]. Countries should therefore adopt effective and scalable approaches for surveillance and strategically deploy these across various epidemiological strata. With respect to malaria vectors, improved surveillance may include measures to better understand the dynamics and pathogen transmission activity of *Anopheles* mosquitoes, as well as measures to monitor insecticide resistance and assess the performance of key interventions such as ITNs and IRS [4].

Unfortunately, most countries still lack adequate capacity for vector surveillance and intervention monitoring [5]. Moreover, current vector control methods are greatly threatened by multiple factors notably insecticide resistance [6, 7], human and mosquito behavioral factors [8–10], and limited durability of ITNs [11], among others. Furthermore, African malaria vectors express varied ecological and biological traits, making their detailed surveillance challenging yet critical to optimize control. For example, understanding the mosquito blood-feeding preferences may illuminate the degree to which certain species can carry human pathogens[12]. Similarly, knowing the age structure of mosquito populations can inform evaluations of the impact of vector control programmes [13], since mosquitoes must attain a certain age to allow maturation of the malaria parasite inside their guts. *Plasmodium falciparum* generally requires > 10 days incubation period inside their vectors before they become infectious [14–16].

Mosquito age-grading previously relied on ovary dissections [13] or, in a few instances, the use of transcriptional profiling [17]. However, these techniques are laborious, subjective, and not optimal for field settings [18]. Emerging techniques in spectroscopy have been considered to address these limitations since they can be performed quickly in dry laboratories without expensive reagents or replacement parts [19] compared to alternatives such as polymerase chain reaction (PCR). Both near-infrared spectroscopy (NIRS) and mid-infrared spectroscopy (MIRS) have been demonstrated to effectively distinguish between mosquito species based on their biochemical components such as proteins, lipids, and carbohydrates [20, 21]. The techniques have also been used for other entomological assessments such as mosquito age-grading [19, 20, 22], studying blood-feeding histories [23], and detection of pathogen infections in mosquitoes [24].

However, the techniques are still in early-stage implementation and are currently operational on a small scale, with no standardized clear guidelines on how to handle mosquito samples for infrared procedures. Previous applications of MIR spectrometer used mosquitoes which were mostly preserved by drying on silica gel (silicon dioxide) prior to scanning [20, 23, 25, 26]. However, past studies that have investigated the influence of mosquito physiological states using NIRS-specific approaches [27] have shown that the technique can be used with mosquito samples preserved using different methods including RNA*later*® (Ambion, Inc., Austin, TX), ethanol, Carnoy's solution, or refrigeration [28, 29]. Other methods for preserving mosquito samples include freezing, DNA-RNA shield, and liquid nitrogen [28, 30, 31]. Most of these preservation methods have not been tested on MIRS-based mosquito applications, and thus it is not known whether variations in preservation methods might affect the performance of these techniques. Therefore, the usage of these preservation methods should be expanded to investigate their feasibility in mid-infrared spectroscopy (MIRS) applications and should possibly also be tested on field-collected mosquitoes, which may have greater ecological and biological variability.

Therefore, the objective of this study was to investigate the effects of different preservation methods on the performance of a previously implemented mid-infrared-based approach for age-grading female malaria vectors. In addition, the study evaluated whether the period of storage duration could influence the performance of age-grading techniques.

## Methods

### Mosquitoes

Laboratory-reared *An. arabiensis* females were used in this study. Larvae were reared in plastic basins and fed on Tetramin® fish food (Tetra GmbH, Melle, Germany). Pupae were then collected from the larval trays and moved into a cage for emergence. Adult mosquitoes were maintained in standard insectary conditions (27 ± 1 °C, 70% relative humidity and a 12 h: 12 h light–dark cycle) at the Ifakara Health Institute’s vector biology laboratory, the VectorSphere. They were fed on 10% glucose solution but not blood and were sampled at the ages of 5 and 17 days old post-emergence to constitute two distinct age classes of young and old mosquitoes. A total of 3681 mosquitoes were used, including 1840 that were 5 days old and 1841 that were 17 days old.

### Preservation and storage

**Fig. 1:**
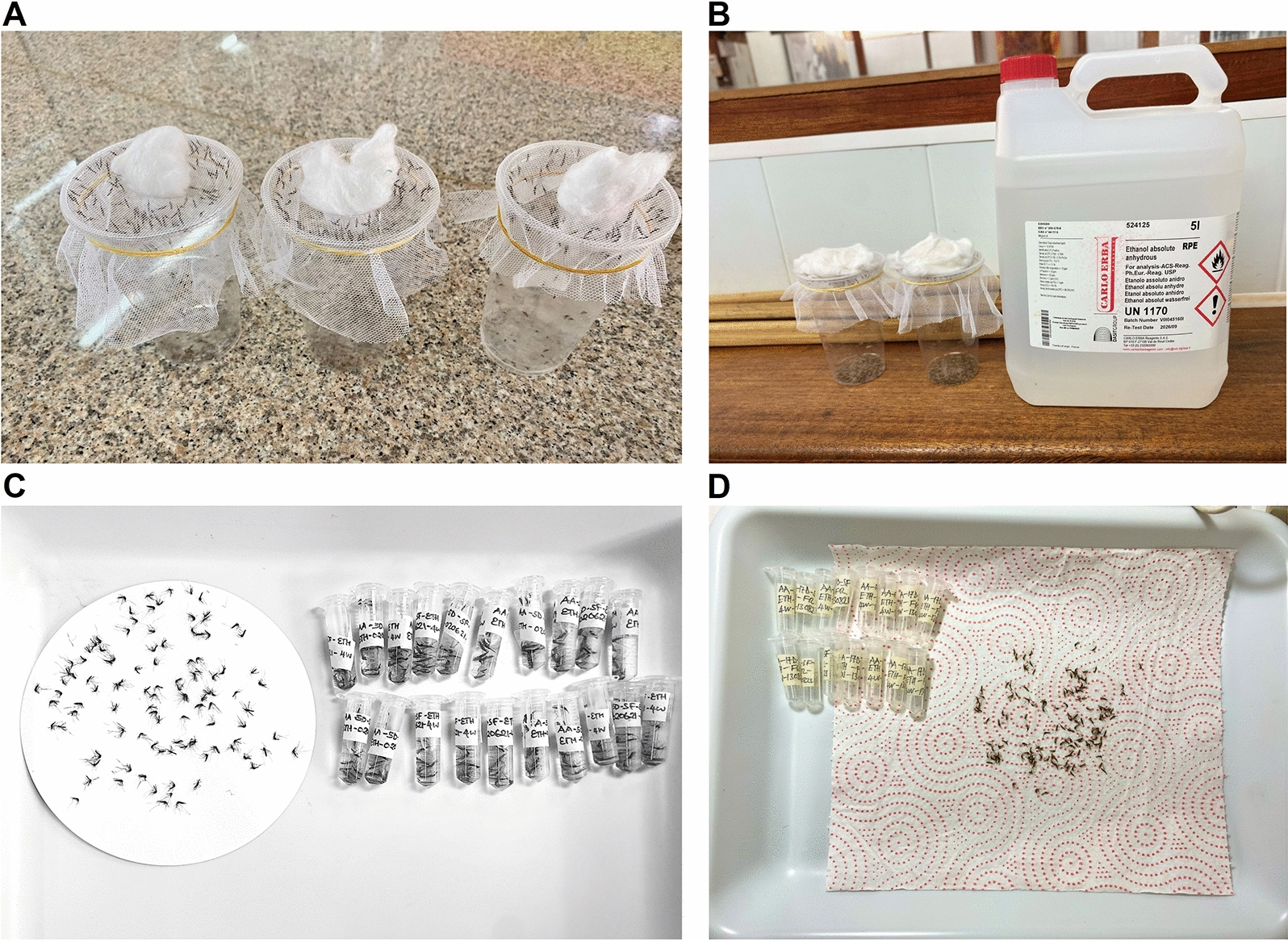
**A** Mosquitoes collected in disposable cups ready to be killed. **B** Mosquitoes anesthetized and killed with ethanol. **C** Mosquito samples being packed in 2-ml Eppendorf tubes ready to be stored for different durations. **D** Mosquitoes placed on paper towels to allow total evaporation of liquid before scanning

Upon collection, mosquitoes were anesthetized and killed using absolute ethanol (Fig. 1). The mosquitoes of each age category (5 and 17 days old) were immediately packed in pools of ten in 2-ml micro-centrifuge tubes and then preserved separately using three different techniques: (i) silica gel desiccation at 5 °C temperature (*n* = 1231), (ii) freezing at − 20 °C (*n* = 1226), or (iii) absolute ethanol at room temperature (*n* = 1224). Desiccation over silica, being the method most commonly used by the research team in previous entomological studies, was considered as the reference. The samples preserved using this procedure were kept at 5 °C to avoid excessive drying, as brittle specimens can be difficult to handle between the ATR crystal and anvil during the scanning process. For each preservation method, mosquitoes were divided into three groups and stored for 1, 4, or 8 weeks separately before being scanned (Table 1).

**Table 1:** Number of mosquitoes scanned for each age, preservation method, and storage duration

Preservation method	No. mosquitoes scanned		Storage duration	Storage temperature
	5 days old	17 days old		
Silica gel	203	204	1 Week	5 °C
	208	208	4 Weeks	
	204	204	8 Weeks	
Ethanol (100%)	204	204	1 Week	26 °C
	203	203	4 Weeks	
	205	205	8 Weeks	
Freezing	204	204	1 Week	–20 °C
	204	204	4 Weeks	
	205	205	8 Weeks	

### Mosquito scanning

The heads and thoraces of the individual mosquitoes were scanned using attenuated total reflection-Fourier transform infrared (ATR-FTIR) ALPHA II spectrometer (Bruker Optics), as previously described [20, 23, 25]. The mid-infrared spectra were recorded at 4000 cm^−1^ to 400 cm^−1^ frequencies with a spectral resolution of 2 cm^−1^. To have noise-free spectra and optimize performance of the spectrometer, 32 background scans were performed without a sample, and the procedure was repeated after every 20 individual sample scans.

Scanning of the mosquito samples was done at the VectorSphere, where the spectrometer is installed. Before scanning, ethanol-preserved samples were placed on paper towels to allow evaporation of the liquid (Fig. 1). The procedure was also performed on mosquito samples that had been frozen to allow the moisture to evaporate. The proprietary Bruker-OPUS software version 7.5 was used to record and process the MIR spectra. At least 203 mosquitoes were used for each age (5 and 17 days old), each storage duration (1, 4, and 8 weeks), and each preservation method (silica, freezing, and ethanol (Table 1).

### Data analysis

The spectral data were first pre-processed by eliminating bands with low intensity, atmospheric water, and CO_2_ interference by using a custom algorithm written in Python 3.8, as previously described by Siria et al. [20]. During this pre-processing, up to 21 individual spectra, which either had significant atmospheric interference from water (H_2_0) and carbon dioxide (CO_2_) or abnormal spectral background noise, were discarded from the main dataset as previously described [23, 26]. The remaining 3660 spectra (1823 from 5-day-old mosquitoes and 1837 from 17-day-old mosquitoes) were further analyzed in Python version 3.8 using *Scikit-learn* version 0.23.2. The corresponding plots and visualizations were done using Seaborn version 0.11 and Matplotlib version 3.3.2. Supervised machine learning approaches were used to train and predict the age of *An. arabiensis* preserved with different preservation techniques and stored for different durations. The intensities of the MIR wavelengths were passed as a matrix of features, and the mosquito ages (5 and 17 days) were used as labels. Features in the spectra dataset were rescaled to have a mean of 0 and a standard deviation of 1, bringing them into a similar scale without distorting the variations in the range of values. Seven machine learning models were evaluated, and the one most suited for mosquito age classification was identified by comparing the baseline accuracies.

The evaluated classifiers included: (i) K-nearest neighbors (KNN), (ii) logistic regression (LR), (iii) support vector machine (SVM), (iv) random forest (RF), (v) gradient boosting (GB), (vi) extra-trees classifier (ET), and (vii) bagging classifier (BGC). Grid search cross-validation was used to further optimize the best-performing algorithm by tuning its hyper-parameters. To evaluate and estimate the performance of the models on unseen data and avoid the risk of over-fitting, the training and test sets were iterated using *K-fold* cross-validation/rotational estimation. For each analysis, the data were a sub-set, so that 80% was used to train the models and the other 20% used as unseen data to evaluate the performance of the models. We combined data from all storage durations (1, 4, and 8 weeks) for each preservation method when evaluating the influence of different preservation techniques on MIR-based age classifications. Similarly, data from all preservation methods (silica gel, freezing, and ethanol) for each storage time were combined when assessing the effects of storage duration on the age classifications.

Models trained with samples preserved using a particular method were evaluated for predicting the age of samples preserved using the same method as well as for predicting the age of samples preserved using other methods. Similarly, models trained on samples stored for 1 week were tested for age-classifying samples kept for the same duration or longer periods (4 or 8 weeks).

## Results

### Effect of preservation methods

**Fig. 2:**
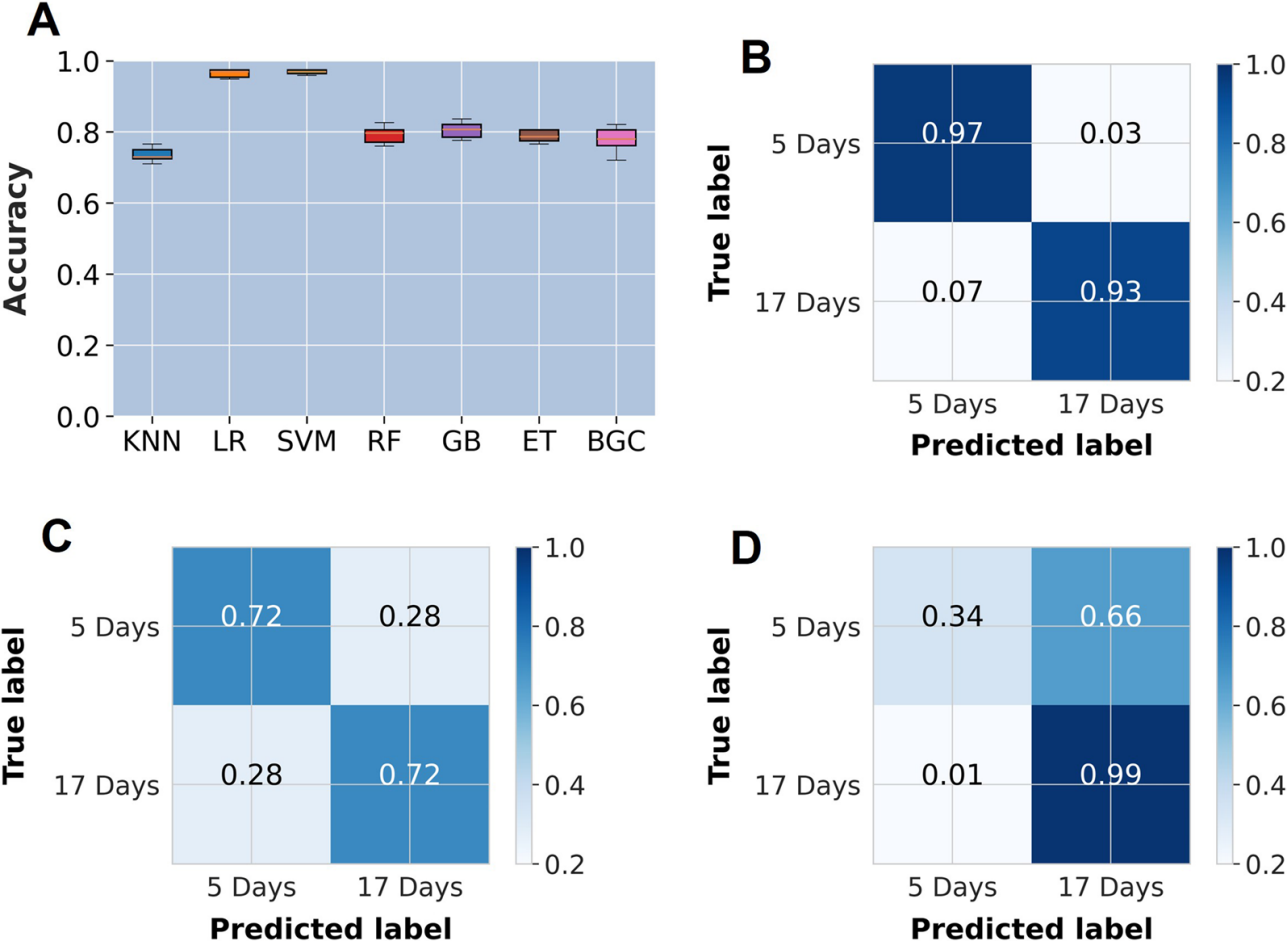
**A** Evaluation of different machine learning classifiers for predicting age for mosquito samples preserved in silica gel. The other three panels show confusion matrices with mosquito age predictions from an SVM classifier trained with silica-preserved mosquitoes and used to evaluate samples preserved in **B** silica gel, **C** ethanol, and **D** freezing

**Fig. 3:**
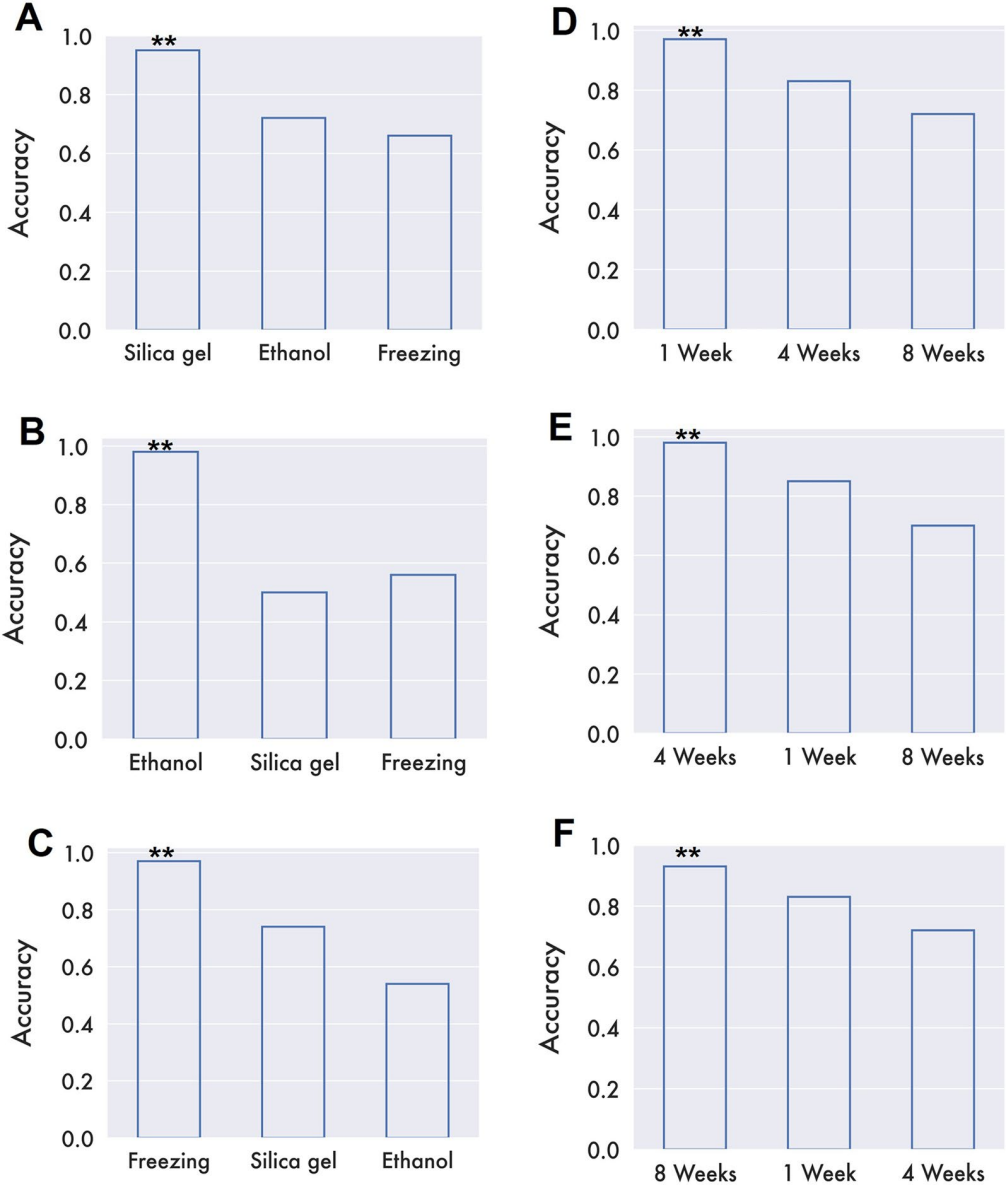
Bar plots showing the declines in classification accuracies when test and training datasets are handled similarly or differently. Here, the SVM models are trained with mid-infrared spectra of mosquitoes preserved using silica (**A**), ethanol (**B**), or freezing (**C**) and then used to predict age classes of samples preserved by one of the three methods. The figure also shows results of the SVM models trained with mid-infrared spectra of mosquitoes stored for 1 week (**D**), 4 weeks (**E**), or 8 weeks (**F**) and then used to predict ages of samples stored for either of the three durations. Reference samples are marked with asterisks. In all cases, the classification accuracy was highest when the training and test samples were handled the same way

The best performing model for predicting the ages of mosquitoes preserved by different methods was the support vector machines classifier (SVM) (Fig. 2). When the SVM model was trained using data of mosquito samples preserved in silica gel, it performed best when predicting the ages of the mosquitoes preserved using the same method achieving a classification accuracy of 95% on the unseen data, whereas the accuracy declined to 72% and 66% when the model was used to classify samples preserved in either ethanol or freezing, respectively. Changes in accuracies were observed when the training set was changed from silica to ethanol, which attained a predictive accuracy of 98% for samples preserved in the same way, 50% for silica gel, and 56% for samples preserved by freezing (Fig. 3). Similarly, when the model was used on samples preserved using freezing, we observed an accuracy of 97% when the model was used to predict frozen samples which declined to 74% for silica and 54% for ethanol-preserved samples (Fig. 3).

### Effect of storage duration

**Fig. 4:**
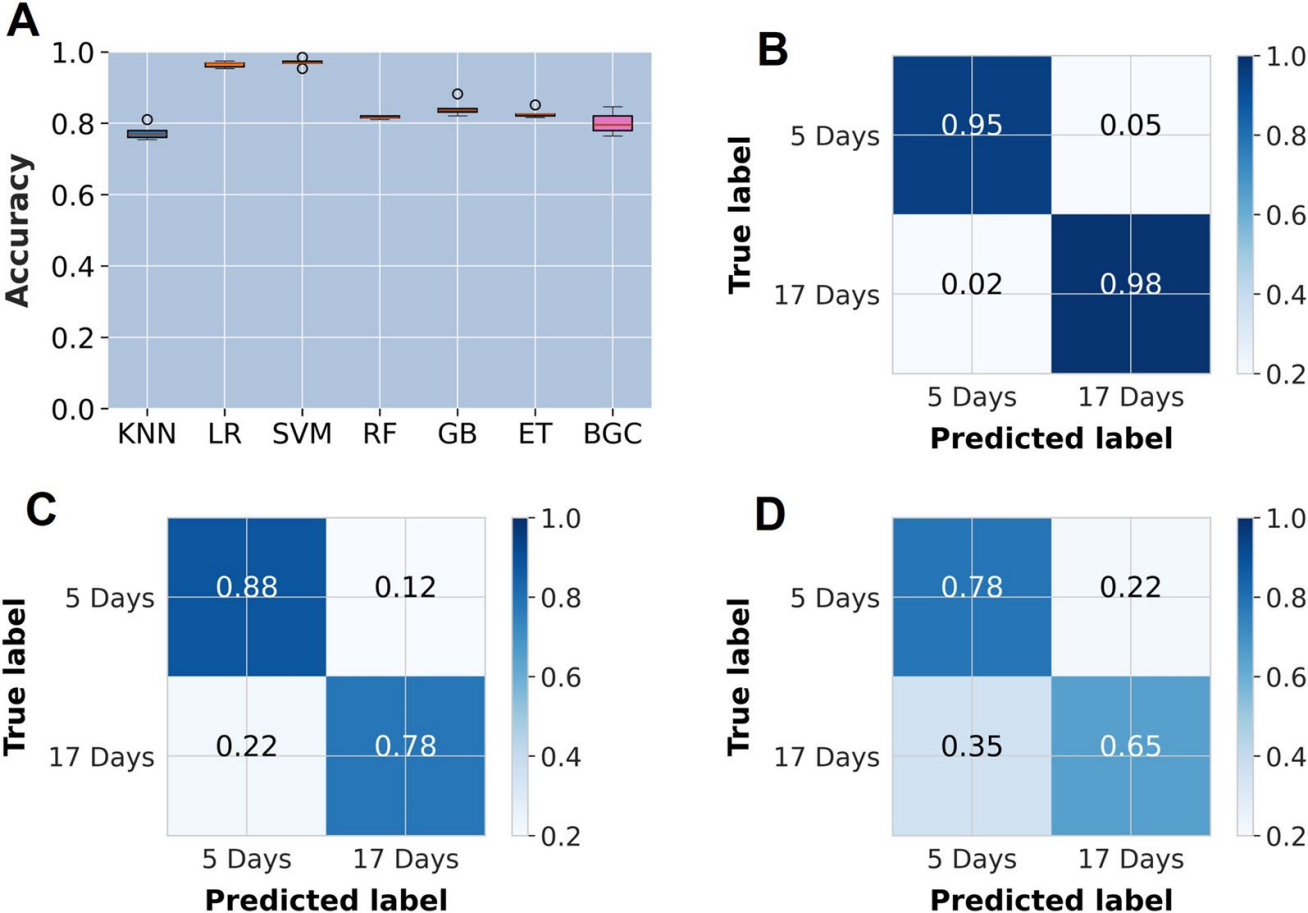
**A** Evaluation of different machine learning classifiers for predicting age of mosquito samples stored for 1 week. The other three panels show confusion matrices with prediction of mosquito ages from an SVM classifier trained with 1-week samples and used to evaluate samples stored for 1 week (**B**), 4 weeks (**C**), and 8 weeks (**D**)

SVM was again the best performing of the seven machine learning classifiers for determining the age of mosquitoes stored for varied time periods (Fig. 4). When trained with data from mosquitoes stored for 1 week and used to predict the age of mosquitoes stored for the same period, the accuracy was 97%. However, the performance deteriorated with an increase in storage duration, such that the accuracies for stored samples were 83% for 4 weeks and 72% for 8 weeks (Fig. 3). When the training set was changed from 1 to 4 weeks storage duration, the accuracy was 98% for 4 weeks, 85% for 1 week, and 70% for 8 weeks. A similar trend was observed when the model was trained with 8-week stored samples achieving an accuracy of 93% (8 weeks), 83% (1 week), and 72% (4 weeks) (Fig. 3).

### Using mosquitoes preserved in silica and stored for 1 week as a reference

**Fig. 5:**
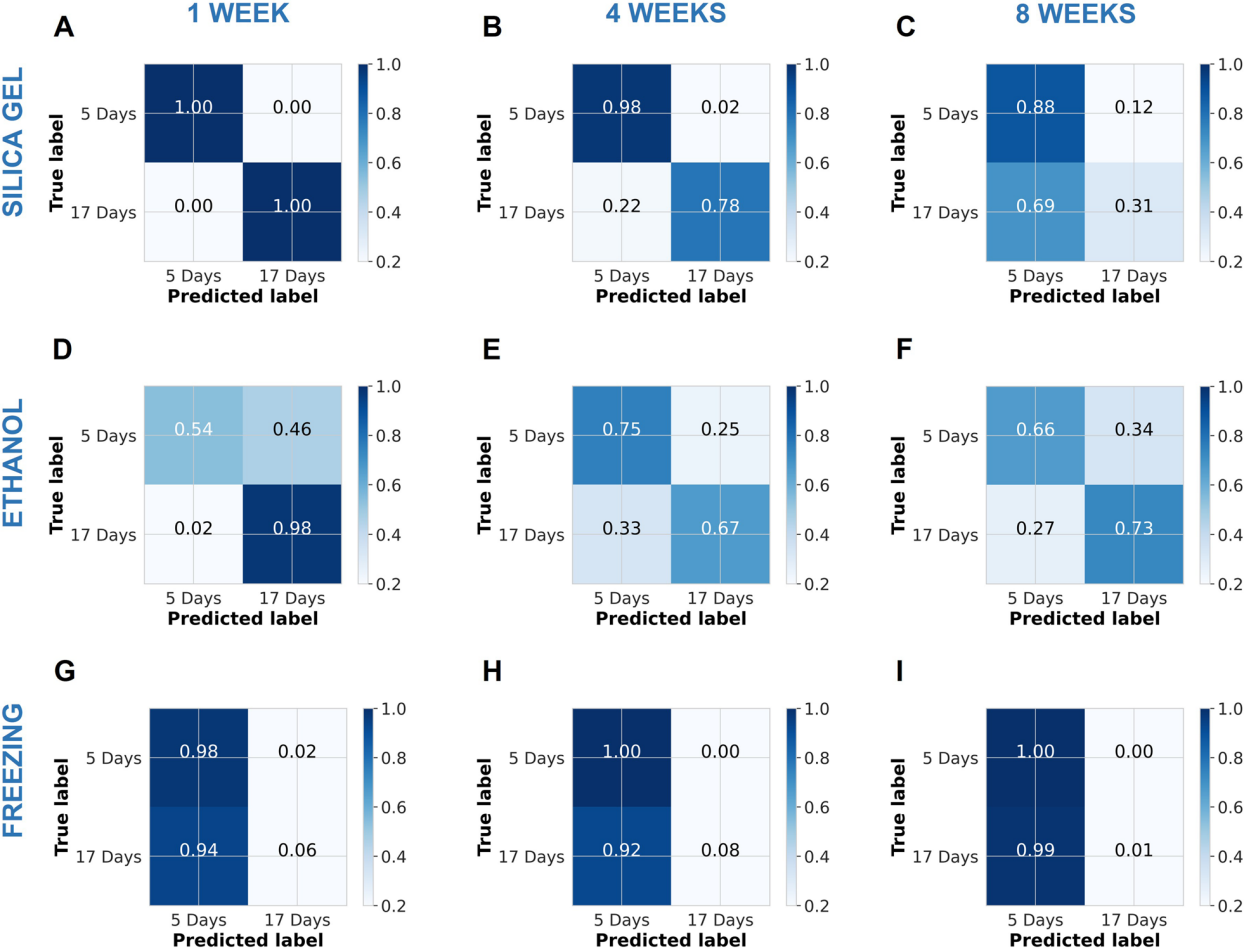
Confusion matrices showing prediction accuracies of mosquito ages from a standard SVM classifier trained with samples preserved in silica gel, stored for 1 week, and then used to predict age classes of test samples handled the same way or differently. Silica-preserved samples are shown in panels** A**,** B**,** C**; ethanol-preserved samples on panels** D**,** E**,** F** and frozen samples on panels** D**,** H**,** I**

Here, mosquitoes preserved for 1 week using silica were used to train an SVM classifier to predict the ages of other mosquitoes preserved by different methods (silica gel, ethanol, or freezing) and stored for different durations (1, 4, and 8 weeks). Table 2 summarizes the data from all the nine tests performed. The resulting classification accuracies varied greatly and were the highest for mosquitoes that had been handled the same way [i.e. 1-week storage in silica (Fig. 5)]. Overall, mosquitoes stored in silica gel generally had the highest classification accuracy up to 4 weeks, whereas mosquitoes stored by freezing had the lowest classification accuracies (Table 2). A decline in prediction accuracies was observed for samples stored in ethanol from 1 to 8 weeks.

**Table 2:** Classification accuracies of a standardized support vector machine (SVM) model trained using mid-infrared spectra from mosquitoes preserved on silica desiccant, stored for 1 week, and used to age-classify other mosquitoes handled in same or alternative ways

Preservation method	Storage duration	Classification accuracy
Silica gel	1 Week	100%
	4 Weeks	88%
	8 Weeks	61%
Ethanol (100%)	1 Week	76%
	4 Weeks	71%
	8 Weeks	70%
Freezing − 20 °C	1 Week	52%
	4 Weeks	54%
	8 Weeks	51%

The age classification accuracies for 5- and 17-day-old mosquitoes preserved using different methods and stored for different durations are summarized in the confusion matrix (Fig. 5).

## Discussion

Infrared-based techniques are increasingly being used for entomological studies such as age grading and species identification of malaria vectors [19, 21, 27, 30–32]. As these techniques are still operational at a small scale, researchers have mostly depended on specific sample handling approaches, with limited considerations for either standardization or alternatives. However, for NIR-specific uses, it has been demonstrated that the methods can work with mosquitoes preserved using multiple techniques. Dowell et al., for example, demonstrated that the chronological ages of mosquitoes could be predicted from NIR-spectra to within 1.4 days when using desiccants, ethanol, Carnoy's solution, RNA *later®*, or refrigeration [29]. Separately, Sikulu et al. showed that preserving mosquitoes in RNA *later®* reduced the likelihood of misclassifying the age of *Anopheles* mosquitoes, further emphasizing the potential of this preservative [28]. These studies demonstrated the expanded potential for infrared-based applications and provided a basis for additional investigations.

Since the infrared spectroscopy applications now increasingly also include the mid-infrared spectral range, studies on different sample handling techniques should be expanded to the MIR application as well. This study investigated the effects of different preservation methods and tested whether different sample storage durations could influence the performance of the age-grading models using laboratory-reared mosquitoes that were either young (5 days old) or old (17 days old), based on mid-infrared spectra.

Desiccation over silica gel is a common preservation method used by entomologists and has also been widely used in previous infrared-based studies; thus, it was considered in this study as the primary reference against which other methods were compared. Similarly, the storage durations of 1, 4, and 8 weeks were selected to represent a practical range over which samples would normally be stored before analysis even in cases where there are limited analytical resources. One week was considered as the baseline against which other durations could be evaluated.

Of the seven classification models tested, support vector machine (SVM) was the best performing at predicting the ages of mosquitoes preserved in different preservation methods, achieving about 96% accuracy. Broadly, the MIRS-based approach could accurately classify the age groups of mosquitoes even after 8 weeks of storage, even though the performance was best for samples stored for 1 week. When data were put together for all storage durations, and the SVM model trained using just samples preserved in silica, the highest accuracy was obtained when the unseen data being predicted were also from silica-preserved mosquitoes. However, when this model was used to predict samples preserved in other methods, the accuracy declined significantly, suggesting the need to standardize the preservation method. The same observation was made when the model was trained using samples preserved by silica for 1 week and then used to perform the predictions for other preservations and storage durations. Here too, the highest predictions accuracies were obtained from the preservation method and storage duration used as a reference (Table 2).

While these data do not necessarily offer a comprehensive analysis of all possible preservation methods, they clearly demonstrate the need to either standardize the preservation methods or at least deploy an additional layer of statistical procedures such as transfer learning [32], where a small amount of different data is introduced into the training set, to neutralize the differences introduced by using different preservatives. Such statistical approaches have been applied to improve predictions on samples collected in different countries or laboratories for mosquito age-grading [20]. In the study conducted by Siria et al., the transfer-learning approach was demonstrably effective at extending the utility of the deep learning models to predict the ages of field-collected mosquitoes in both Tanzania and Burkina Faso [20]. Perhaps the most practical option would simply be to require standardized treatments of samples in both laboratory and field studies.

Silica gel has been used by researchers in preserving many samples for species identification, age-grading, and blood-meal experiments at a relatively low cost [20, 23, 25, 33]. This makes it ideal for storing large numbers of mosquito samples in field settings over long durations. This current study has also shown that silica gel desiccation may be ideal for storing mosquito samples for a short period at ~ 5 °C, as these were the samples for which age classification was most accurate. Contrarily, frozen samples achieved far lower age predictions across the different storage durations, when the SVM model was trained on silica gel as a reference (Table 2). These samples were not as dry as those preserved in silica gel for the same duration; therefore, excess water content may have limited the full potential of the machine learning predictions of the spectral data even after the data were cleaned. Because of the moisture content even after the drying period before scanning, the frozen samples were easily crushed by the anvil and provided no resistance when pressed against the ATR crystal of the spectrometer. This may add additional complexity to data analysis, requiring that certain data points are discarded because of excessive water content as previously suggested by González et al. [26]. Lastly, ethanol has also been used widely for mosquito preservation, especially where the nucleic acid component is needed for further analyses [34]. In this study, the prediction accuracies of models trained with ethanol-preserved samples dropped to 50% when age-classifying silica-preserved mosquitoes and to 56% when age-classifying frozen samples. Further analysis, beyond the scope of this current study, may be needed to evaluate these comparisons, and possibly include additional statistical approaches.

This study also allowed direct assessment of whether variations in storage duration can impact the accuracy of mid-infrared-based approaches for mosquito age-grading. Here, the samples stored for 1 week were initially used as the standard reference and used to train the basic machine learning models, which were then used to predict the age of mosquitoes stored for different durations. It is particularly important to evaluate these differences since entomological surveys are typically time-consuming and can generate very large numbers of samples that cannot be analyzed immediately on the same day. As a result, some form of extended storage for weeks or months is often necessary, especially where the equipment for sample analysis does not exist on-site.

Overall, these results suggest that mid-infrared spectroscopy coupled with machine learning can predict the age of mosquito species stored in different preservation methods for different periods of up to 8 weeks. As previously demonstrated, the approach has the advantages of being quick to perform, cost-effective, and reagent-free, this being reliable even in low-resource settings [20, 26].

One limitation of this study was that it was carried out for only one species of malaria vector, and therefore more research is needed to investigate whether there will be any variations in other malaria-transmitting mosquito species. Also, the mosquitoes used in this study were not blood-fed and therefore did not fully simulate the natural mosquito life cycle processes. To further reduce experimental variations, the experiments used only two mosquito ages (5 and 17 days old). These factors could be addressed in future studies by expanding the range of ages and physiological states of mosquitoes so as to be more representative of the natural world. It may also be necessary to evaluate silica-preserved mosquitoes without any refrigeration as done in this study, since it will not be operationally feasible for large-scale field studies and areas with limited or no access to electricity. Moreover, the range of preservatives and durations of storage were limited to just three each to ensure feasibility. Further investigation may reveal that such models may respond differently when using an expanded range of preservatives or storage durations. Nonetheless, the data show that silica-based preservation is a satisfactory starting point for samples destined for spectroscopy and can be used for several weeks of storage.

## Conclusion

This study has demonstrated that both the preservation methods and storage durations are important determinants of the classification accuracy used to predict mosquito ages using mid-infrared spectra data. Furthermore, we observed that the highest accuracies are achieved when the training samples are preserved the same way and stored for the same duration as the test samples. Additionally, among all the preservation methods used, drying over silica gel was the best method and could be used for up to several weeks. Protocols for entomological studies should therefore specify the need to standardize sample-handling procedures for infrared-based approaches. Alternatively, additional machine learning techniques such as transfer learning and deep learning approaches may be incorporated to improve prediction accuracy between distinct groups.

## Data Availability

All data for this study will be available upon request. The datasets and scripts used to support the findings of this work are accessible in a GitHub repository at https://github.com/MwangaEP/Wellcome-fellowship

## Abbreviations

MIRS: Mid-infrared spectroscopy
ML: Machine learning
ATR-FTIR: Attenuated total reflectance-Fourier transform infrared spectrometer
NIR: Near-infrared
KNN: K-nearest neighbors (KNN)
LR: Logistic regression
SVM: Support vector machine
RF: Random forest
GB: Gradient boosting
ET: Extra-trees classifier
BGC: Bagging classifier
